# Hsa_circ_0054633 association of C peptide is related to IL‐17 and TNF‐α in patients with diabetes mellitus receiving insulin treatment

**DOI:** 10.1002/jcla.23856

**Published:** 2021-07-17

**Authors:** Huixue Dai, Fei Hu, Xiangwei Yang, Peng Hu, Yudong Chu, Shizhong Bu

**Affiliations:** ^1^ Department of endocrinology Ninghai Chengguan Hospital Ningbo China; ^2^ Diabetes Research Center School of Medicine Ningbo University Ningbo China; ^3^ Zhejiang Provincial Key Laboratory of Pathophysiology Department of Biochemistry and Molecular Biology School of Medicine Ningbo University Ningbo China; ^4^ Cixi Biomedical Research Institute Wenzhou Medical University Cixi China; ^5^ Department of Nephrology Ningbo Medical Center Lihuili Hospital Ningbo China

**Keywords:** C peptide, Hsa_circ_0054633, IL‐17, TNF‐α, type 2 diabetes mellitus

## Abstract

**Background:**

Chronic inflammation damaged the islet and resulted in dysfunction of T2D. Circular RNA is stable and better for biomarker in many diseases. Here, we aimed to identify potential circular RNA hsa_circ_0054633 that can be a biomarkers for the effects of insulin therapy in T2D.

**Methods:**

In this retrospective case‐control study, patients were from Li Huili Hospital, Ningbo, China, from February 10, 2019, to August 15, 2019. We included 47 healthy adults, 46 new‐onset T2D with insulin resistance, and 51 patients with insulin therapy. Serum inflammation factors were tested by ELISA assays. We selected hsa_circ_0054633 as a candidate biomarker and measured its concentration in serum by qRT‐PCR. The Pearson correlation test was used to evaluate the correlation between this circRNA and clinical variables.

**Results:**

Clinical data indicated that serum C peptide was increased in T2D treatment with insulin. Serum hsa_circ_0054633 was decreased in insulin treatment group. Hsa_circ_0054633 was negative correlated with C peptide (*r* = −0.2841, *p* = 0.0433,). IL‐1 and IL‐6, IL‐17, and TNF‐α were higher in T2D patients and decreased after insulin treatment, only IL‐17 and TNF‐α showed a positive correlation to hsa_circ_0054633 (*r* = 0.4825, *p *< 0.0001, and *r* = 0.6190, *p *< 0.0001). The area under ROC curve was 0.7432, 0.5839, and 0.7573 for Hsa_circ_0054633, C peptide, and their combination.

**Conclusion:**

Hsa_circ_0054633 level was lower in T2D with insulin treatment than untreated and was a negative correlation with C peptide, and positively correlated with IL‐17 and TNF‐α, suggesting that hsa_circ_0054633 may be a potential early indicator of insulin treatment effect to improve inflammation condition.

## Graphical abstract

Hsa_circRNA_0054633 was lower expression in T2D with insulin therapy. There was no significantly change between normal group and T2D without insulin therapy group (*p *> 0.05, T2D without insulin treatment vs. normal group). The level of Hsa_circRNA_0054633 in T2D patients with insulin treatment was marked lower in T2D treated with insulin than in without insulin (*p *< 0.05, vs. T2D without insulin treatment).

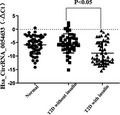

## 1 INTRODUCTION

Type 2 diabetes (T2D) is characterized by chronic inflammation condition,^1^, ^2^, ^3^ this concept is a helpful and novel way to understand this disease and has implications for the pathogenesis and the complications of this disease. However, its mechanisms are still not fully understood. Interleukin (IL)‐1 is a major player in the regulation of inflammatory processes. Ongoing clinical trials will address the probably of IL‐1 antagonism to prevent diabetes and other related complications.^4^ Interleukin 6 (IL‐6), diversity functions of cytokine, has been indicated in the clinical pathophysiology of T2D. High circulating level of IL‐6 is an independent predictor of T2D and is deemed to be involved in the development of inflammation, insulin resistance, and β‐cell dysfunction.^5^ Interleukin‐17 (IL‐17) is produced by CD8^+^CD49a^−^ tissue‐resident memory T (Trm) cells. Over‐production of L‐17 promotes autoimmune diseases,^6^ moreover, IL‐17 has been considered in regulating body fat, which is exceedingly relevant incidence increase in obesity and type 2 diabetes.^7^ Tumor necrosis factor‐alpha (TNF‐α) is a critical pro‐inflammatory intervenor that is climatically involved in the development of insulin resistance and pathogenesis of T2D.^8^ The high TNF‐α level is a critical common characteristic in insulin resistance in adipocytes and peripheral tissues by destroying the insulin signaling that resulting in the development of T2D. Whereas, anti‐TNF‐α therapy plans have been reduced the incidence of insulin resistance and development of T2D.^9^

Insulin resistance (IR) is a physiological condition related to type 2 diabetes mellitus (T2DM) and obesity, which is also associated with high blood insulin and glucose.^10^, ^11^ IR is an overcompensated secretion of insulin caused by decreased insulin uptake and glucose utilization efficiency, which leads to the pathological manifestations of hyperinsulinemia.^10^, ^12^ IR has many risk factors. Diets, genetic factors, and environmental factors can lead to decreases in insulin uptake and glucose utilization rate, which then result in IR.^13^

Gene transcripts without protein‐coding potential are referred to as non‐coding RNAs (ncRNAs). Circular RNA (circRNA) is widely found in various organisms.^14^ Unlike normal linear RNA, a circRNA’s 3’ end is linked to the 5’ end to form a covalently closed‐loop structure, which makes it more stable and conserved than linear RNAs.^14^ Moreover, circRNA may be used as potential biomarkers to diagnose and predict diseases such as cancer, cardiovascular disease, endocrine and metabolic diseases, and central nervous system diseases.^15^, ^16^, ^17^ Zhao et al. used a gene chip to analyze peripheral blood samples from patients with type 2 diabetes. They found 489 circRNAs to be differentially expressed in these patients relative to healthy controls, of which 78 were upregulated and 411 were downregulated and proposed that hsa_‐_circ_‐_0054633 can be used as a diagnostic marker to evaluate pre‐diabetes or T2D.^18^ Yan et al.^19^ used sequencing technology to analyze placental villus tissue in patients with gestational diabetes and identified 227 upregulated circRNAs and 255 downregulated circRNAs. Shang et al. performed RNA sequencing analysis on high glucose‐treated human endothelial cells and found 95 differentially expressed circRNAs.^20^ CircRNA has been reported to be involved in the regulation of insulin secretion and the pathogenesis of diabetes. CircRNA‐CDR1 is a natural antisense transcript of CDR1, which has been found to regulate insulin secretion and β‐cell renewal,^21^ but the relationship between cirRNA and IR has not been well studied. Wu et al. reported that hsa_circ_0054633 is highly expressed in gestational diabetes and is closely related to the glycosylation index.^22^ Zhao et al. discovered that hsa_circ_0054633 in peripheral blood can be used as a diagnostic biomarker for pre‐diabetes and T2D.^18^ The purpose of our research is to test whether hsa_‐_circ_‐_0054633 can be used as an indicator of the effects of insulin therapy.

## 2 MATERIALS AND METHODS

### 2.1 Subjects

A case‐control study was conducted at the Ningbo University Medical Center Li Huili Eastern Hospital, Zhejiang, China. The time the patients were diagnosed with diabetes was from February 10, 2019, to August 15, 2019. Subjects included healthy controls and patients with IR (insulin therapy and no insulin therapy). We excluded patients with diabetes and other comorbidities, such as hypertension, hepatorenal syndrome, or cancer. Endorsed by the Ethics Committee of the Ningbo University School of Medicine. During the collection of specimens for this study, all participants provided informed consent to use their blood samples in research. The diagnostic criteria for diabetes were any of the following three conditions: typical symptoms of diabetes (increased thirst, increased urine, increased eating, and weight loss); random blood glucose ≥11.1 mmol/L or FPG ≥7.0 mmol/L (125 mg/dl), where fasting is defined as no caloric intake for at least 8 h; or OGTT (oral glucose tolerance test) 2 h PG ≥11.1 mmol/L (200 mg/dl). The standard for IR was the clinically commonly used oral glucose tolerance and insulin release test (HOMA‐IR), HOMA‐IR = fasting blood glucose (FPG) × fasting insulin / 22.5. HOMA‐IR >2.69 was diagnosed as IR.

Specimens were collected from 47 normal patients, 46 patients with IR who did not use insulin therapy, and 51 IR with IR who received insulin therapy. Blood biochemistry markers including triglyceride (TG), total cholesterol (TC), low‐density lipoprotein (LDL‐L), and high‐density lipoprotein (HDL‐L), fasting blood glucose (FPG), glycated hemoglobin (HBAIC), insulin secretion, and C peptide were assessed using a chemical analyzer (Siemens). Age and BMI were also recorded.

### 2.2 Specimen collection and preservation

Patient serum samples were collected from the Li Huili Eastern Hospital of Ningbo University Medical Center from February 10, 2019, to August 15, 2019. Peripheral venous blood (3 ml) was collected and centrifuged (3000 rpm for 10 min at room temperature). Serum was then collected immediately and stored at −80℃ until use.

### 2.3 Total RNA extraction and cDNA synthesis

Total RNA was isolated from serum using TRIzol LS reagent (Invitrogen) according to the manufacturer's instructions. The purity of the extracted RNA was measured by an ultraviolet spectrophotometer, and samples with an absorbance ratio of 260/280 nm between 1.8 and 2.1 were used for further analysis.

Using HiFI‐MMLV cDNA First‐Strand Synthesis Kit (CWBIO) at 42℃ for 50 min and 85℃ for 5 min to reverse‐transcribe RNA into cDNA, and immediately store at −80℃ for use.

### 2.4 Quantitative reverse transcription‐polymerase chain reaction (qRT‐PCR)

Hsa_circ_0054633 Primer sequences were Forward:TTGCTTTCTACACTTTCAGGTGAC. Reverse:GCTTTTTGTCTGTAGTCAACCACCA. For quantification, real‐time PCR analysis was performed using a LightCycler 480 SYBR Green I Master kit on a LightCycler 480 II (Roche). qRT‐PCR was performed under the following conditions: 95℃ for a 5 min initial denaturation step, then there's 94 out of 45 cycles °C for 10 s, primer for specific annealing temperature of 20 s, and 72 s °C for 30 s. The annealing temperature of HSa_circ_0054633 is 59℃, and that of β‐actin is 56℃. Relative fold changes were calculated using the threshold cycle method and β‐actin was used as an internal normalization control. The experiment was performed three times independently.

### 2.5 ELISA assays

Serum IL‐1, IL‐6, IL‐17, and TNF‐α were measured by ELISA assays as recommended by the manufacturer. Kits of IL‐1 (BMS2080, Interassay Coefficients of Variability (CV) = 7.4%, Intraassay CV = 7.2%), IL‐6 (EH2IL6, Interassay CV < 10%, Intraassay CV < 10%), TNF‐α (KAC1751, Interassay CV = 6%, Intraassay CV = 3%) were purchased from Invitrogen, CA, US. TNF‐α was purchased from QiaoDu biotec company (CB25701362, Interassay CV < 10%, Intraassay CV < 10%), shanghai, China.

### 2.6 Statistical analyses

Data were performed and statistical analysis using GraphPad Prism 5.0, SPSS software, version 18.0 for Windows. Variables were expressed as means ± standard deviations, percentage. First, all data were tested using the Kolmogorov‐Smirnov test to determine whether the values were normally distributed. When a variable satisfied the normal distribution, the homogeneity test of the variance was performed. An independent sample *t*‐test was used when both the normal distribution and the homogeneity of the variance were satisfied. If one of the conditions was not met, the Mann‐Whitney U‐test is used. When comparing the frequencies of categorical variables, we used Pearson's *χ* ^2^ test. *p *< 0.05 was considered statistically significant. The correlation between two variables was analyzed using Pearson's correlation test.

## 3 RESULTS

### 3.1 Participant characteristics

As shown in Table 1, three groups’ clinical data were compared with one‐way ANOVA. The Bonferroni test method was used for multiple comparisons. Age, body mass index (BMI), SBP, DBP, fasting blood glucose (FPG), glycated hemoglobin (HbA1c), and C peptide were statistically different in each group, but only C peptide was statistically significant difference between insulin unused and used groups, *p *= 0.009.

**TABLE 1:** Clinical characteristics of type 2 dabetes patients

Characteristic	Normal	IR without insulin treatment	IR with insulin therapy	*F*	*p* value^a^
n	47	46	51		
Age, year	66.83 ± 1.43	61.04 ± 1.63	61.63 ± 1.66	3.950	0.021*
Medical history,year	0	0	11.33 ± 0.86		
BMI, Kg/m^2^	23.13 ± 0.20	24.84 ± 0.25	24.65 ± 0.23	16.368	<0.0001****
SBP, mmHg	139.2 ± 1.76/	145.7 ± 1.83/	145.9 ± 1.95	4.155	0.018*
DBP,mmHg	71.32 ± 0.95	80.96 ± 1.40	80.96 ± 1.40	17.169	<0.0001****
FPG, mmol/L	5.30 ± 0.06	8.98 ± 0.29	9.80 ± 0.43	60.210	<0.0001****
HbA1C, %	5.31 ± 0.05	8.54 ± 0.21	9.23 ± 0.23	126.674	<0.0001****
TG, mmol/L	1.97 ± 0.14	2.65.01 ± 0.13	2.23 ± 0.22	2.568	0.080
TC, mmol/L	4.84± 0.12	6 ± 0.27	5.04 ± 0.15	0.619	0.540
HDL‐L, mmol/L	1.26 ± 0.043	1.15± 0.04	1.23 ± 0.05	1.677	0.191
LDL‐L, mmol/L	3.09 ± 0.11	3.05 ± 0.14	2.90 ± 0.13	0.709	0.494
INS, pmol/L	65.79 ± 3.11	141.9 ± 8.56	138.4 ± 9.34	31.222	<0.0001****
C peptide, ng/ml	2.69 ± 0.69	0.49 ± 0.24	0.85 ± 0.25^b^	6.503	0.002***

^b^ vs IR without insulin treatment.

*< 0.05

***< 0.005

****< 0.001

### 3.2 Expression of hsa_circ_0054633 is lower in patients receiving insulin therapy

**FIGURE 1:**
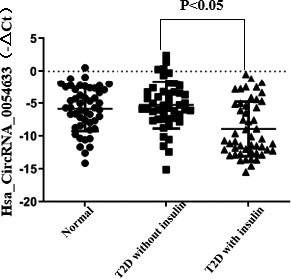
Hsa_circ_0054633 was lower expression in T2D with insulin therapy. There was no significantly change between normal group and T2D without insulin therapy group (*p *> 0.05, T2D without insulin treatment *vs*. normal group). The level of Hsa_circ_0054633 in T2D patients with insulin treatment was marked lower in T2D treated with insulin than in without insulin (*p *< 0.05, vs. T2D without insulin treatment)

By qRT‐PCR, we found that the expression of hsa_circ_0054633 in patients with IR who were not treated with insulin was higher than that in normal people, but the difference was not statistically significant (*p *> 0.05), and the reason needs further study. In addition, we found that the levels of hsa_circ_0054633 in the serum of patients with IR who were treated with insulin were significantly lower than normal group and diabetes without insulin treatment group (*p* = 0.0002 and *p *< 0.0001, Figure 1).

### 3.3 **Hsa_circ_0054633** **level is correlated with the C peptide**

**FIGURE 2:**
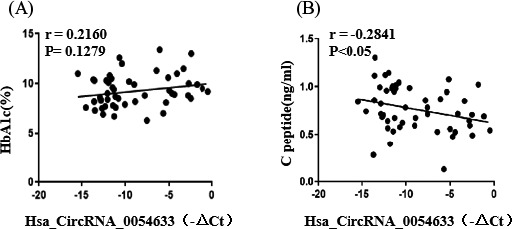
Hsa_circ_0054633 was negative correlated with C peptide. Hsa_circ_0054633 was negative correlated with C peptide (*r* = −0.2841, *p *= 0.0433). There was no significant correlation between hsa_circ_0054633 and HbA1c (*r* = 0.2160, *p *= 0.1279)

BP, HbA1c, and C peptide in patients with insulin resistance were significantly different between those with and without insulin treatment (Table 1). Because BP is caused by poor blood vessel function in the elderly, we chose HBA1c and C peptide as indicators in this study. As shown in Figure 2, we use Pearson's correlation test to analyze the correlation between the hsa_circ_0054633 level and HbA1c and C peptide. The level of hsa_circ_0054633 was correlated with the serum C peptide level (*r* = −0.2841, *p *= 0.0433). However, there was no significant correlation between hsa_circ_0054633 and HbA1c (*r* = 0.2160, *p *= 0.1279).

### 3.4 Insulin therapy in T2D patients improved chronic inflammation factors of IL‐1, IL‐6, IL‐17, and TNF‐α

**FIGURE 3:**
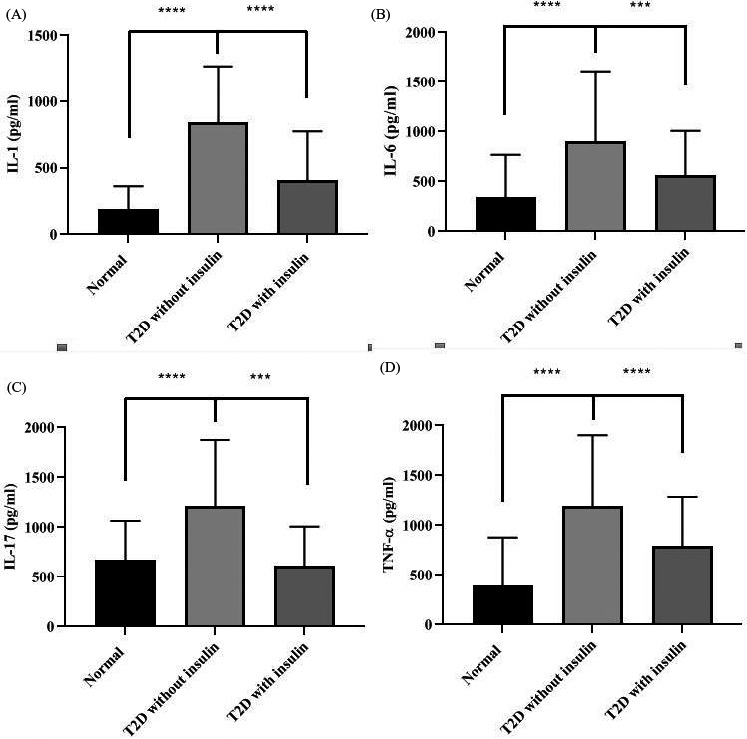
Inflammation factors were high level in serum of T2D patients. (A) IL‐1 in Normal, T2D without insulin and T2D with insulin, IL‐1 level was increased in T2D patients without insulin treatment (*p *< 0.0001 vs. normal group), but significantly decreased after treatment with insulin (*p *< 0.0001 vs. insulin treatment group). (B, C and D) IL‐6, IL‐17, and TNF‐α levels were significantly increased in T2D without insulin treatment groups (all of them, *p *< 0.0001, vs. normal groups), however, IL‐6, IL‐17, and TNF‐α levels were significantly decreased in groups with insulin treaded (in IL‐6 and IL‐17, *p *< 0.0001 and in TNF‐α, *p *< 0.001, all vs. insulin treatment group)

Since chronic inflammation plays a key role in T2D pathogenesis, we next to test the major inflammation factors, for example, IL‐1, IL‐6, IL‐17, and TNF‐α in the serum of each experiment groups by ELISA assays. As showed in Figure 3A, serum IL‐1 level was markedly increased in IR without insulin treatment (*p *< 0.0001), and then was decreased in IR with insulin treatment (*p *< 0.0001). Like IL‐1, serum IL‐6 level was higher in IR without insulin treatment than in normal group (*p *< 0.0001), but was lower in IR diabetes patients with insulin treatment than in those without insulin treatment (*p *< 0.001), as shown in Figure 3B. For IL‐17, which serum level was significantly increased in IR with insulin treatment than in normal group (*p *< 0.0001), and restored almost to the normal group level when treatment with insulin (*p *< 0.0001), showed in Figure 3C. As showed in Figure 3D, similar to other inflammation factors, TNF‐α was obviously increased in IR with insulin treatment than in normal group (*p *< 0.0001), and decreased in group of IR without insulin treatment (*p *< 0.001).

### 3.5 Hsa_circ_0054633 is interrelated with the IL‐17 and TNF‐α but not IL‐1 and IL‐6

**FIGURE 4:**
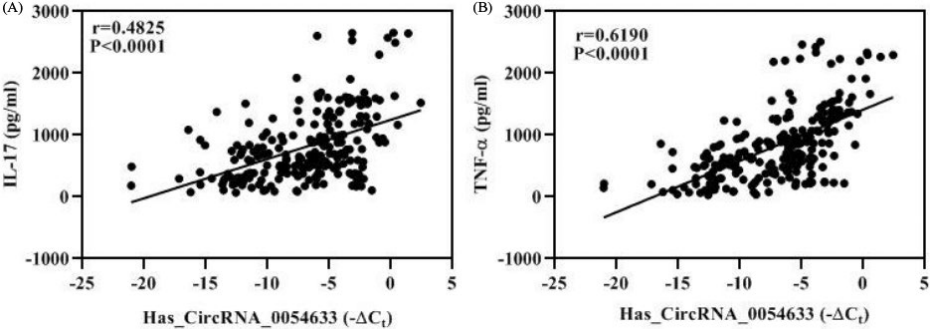
Hsa_circ_0054633 was positive correlation with IL‐17 and TNF‐α. (A) Hsa_circ_0054633 was positive correlation with IL‐17(*r* = 0.4825, *p *< 0.0001). (B) Hsa_circ_0054633 was correlation with TNF‐α (*r* = 0.6190, *p *< 0.0001)

To study whether inflammation factors were associated with Has_ circ_0054633, we analyzed the correlation between this CircRNA with all four indicated inflammation factors by GraphPad Prism for Windows and SPSS software. As Figure 4A, B, Has circ_0054633 was position correlation with IL‐17 (*r* = 0.4825, *p *< 0.0001) and TNF‐α (*r* = 0.6190, *p *< 0.0001). There was no correlation between this CircRNA with IL‐1 and IL‐6, data were not shown.

### 3.6 Potential diagnostic pulse of Hsa_circ_0054633 to islet function

**FIGURE 5:**
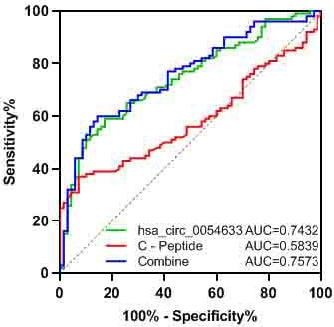
ROC analysis of expression of Hsa_circ_0054633 and serum C peptide level. The ROC curve analysis was conducted, and the area under ROC curve was 0.7432, 0.5839, and 0.7573 for Hsa_circ_0054633, C peptide, and their combination. AUC mean area under the curve

Next, we explored the potential diagnostic value of Hsa_circ_0054633 and C peptide. The ROC curve analysis was conducted, and the area under ROC curve was 0.7432, 0.5839, and 0.7573 for Hsa_circ_0054633, C peptide, and their combination, respectively, in the cohort (Figure 5). For potential diagnostic value, Hsa_circ_0054633 would be better than C peptide and almost the same as the combination of Hsa_circ_0054633 and C peptide.

## 4 DISCUSSION

Insulin resistance is one of the early symptoms and core characteristics of T2D and characterized by a reduced physiological response of target tissues to normal insulin levels and leads to reduced glucose utilization in muscle and fat, as well as increased liver glycogenogenesis.^23^ IR is the result of the combined effects of genetic and environmental factors. Its mechanism of action is very complex. Studies have shown that IR is associated with lipid metabolism disorders, inflammatory factor secretion, oxidative stress, and endoplasmic reticulum stress.^24^

In patients who were treated with insulin, the diastolic blood Serum HbA1c levels represent the response to the blood glucose control treatment in diabetic patients in the past two months.^25^, ^26^ The higher the HbA1c, the poorer the blood glucose control level. Here, we also observed that the HbA1c level in patients with IR was higher than that in the healthy controls. The HbA1c level was high in patients treated with insulin, indicating that the patients’ glycemic control was poor, and additional treatments should be considered. In addition, the FBP was high in the patients with IR receiving insulin treatment, further suggesting that their clinical treatment plan needs to be improved. Pressure improved, but the systolic blood pressure did not improve.

Serum insulin and C peptide levels reflect the reserve function of islet β cells.^27^, ^28^ In the early stage of T2D, serum insulin is normal or increased. With the progression of the disease, pancreatic islet function gradually decreases and insulin secretion capacity decreases. Our study found that serum insulin and C peptide levels in patients with IR who were not treated with insulin were higher than those in normal people.

Recently, more and more researchers have proposed that epigenetic factors such as non‐coding RNA are involved in the regulation of glucose metabolism.^29^, ^30^, ^31^ CircRNA is much more stable in cells than linear RNA. In some tissues, they are expressed at 10 times the level of linear RNA. Therefore, circRNA may be a better biomarker than linear RNA.^32^ We selected has___circ_0054633 as a biomarker and tested it in normal people, patients with IR, and patients with IR treated with insulin. It was found that hsa_circ_0054633 expression was significantly lower in patients treated with insulin than in patients not treated with insulin. Research has shown that hsa_circ_0054633 in peripheral blood can be used as a biomarker for the diagnosis of pre‐diabetes and T2D.^18^ Zhao et al. showed that hsa_circ_0054633 is involved in the cell cycle process and is closely related to molecular catabolism.^18^ In this study, inflammation factors of IL‐1, IL‐6, IL‐17, and TNF‐α were decreased after insulin treatment, but there were no evidence to show insulin could impact inflammation factors in current research. However, insulin therapy controlled hyperglycemia may improve chronic inflammation condition in T2D patients.^33^ Our data indicated that after insulin treatment, C peptide level in T2D patient was increased although not back to normal level. At the same time hsa_circ_0054633 was going down with negative correlation of C peptide change and with positive correlation of IL‐17 and TNF‐α, but not of IL‐1 and IL‐6. As endogenous insulin precursor, C peptide level increasing implied that the function of pancreatic islet β cells recovered.^34^, ^35^ These data suggested hsa_circ_0054633 may be interrelated with inflammation of T2D. The proliferation of pancreatic islet β cells is regulated by the cell cycle process, and decreased insulin secretion caused by reduced β cell proliferation is the main cause of diabetes. IR is due to the decreased efficiency of insulin in promoting glucose uptake and utilization, and the body's compensatory production of excessive insulin produces hyperinsulinemia to maintain blood glucose stability.^36^, ^37^ Therefore, our results suggest that hsa_circ_0054633 may be involved in glucose uptake and utilization in IR. Furthermore, we found a correlation between hsa_circ_0054633 and C peptide, indicating that IR patients treated with insulin had reduced endogenous insulin requirements and increased glucose utilization. Although C peptide is a current Clinical test indicator for islet function, as a potential diagnostic marker, Has_circ_0054633 might be much more values for anticipation of islet function than C peptide and almost the same as their combined in insulin therapy for diabetic patients.

In conclusion, hsa_circ_0054633 level was lower in patients treated with insulin than untreated and was negative correlated with C peptide level. This circRNA was positive correlated with inflammation factors IL‐17 and TNF‐α. These findings suggested that hsa_circ_0054633 might be a potential indicator of the effectiveness of insulin therapy.

## CONFLICT OF INTEREST

No potential conflict of interest was reported by the authors.

## Data Availability

Data are available on request to the authors.
